# Can cloud point-based enrichment, preservation, and detection methods help to bridge gaps in aquatic nanometrology?

**DOI:** 10.1007/s00216-016-9873-5

**Published:** 2016-08-24

**Authors:** Lars Duester, Anne-Lena Fabricius, Sven Jakobtorweihen, Allan Philippe, Florian Weigl, Andreas Wimmer, Michael Schuster, Muhammad Faizan Nazar

**Affiliations:** 1 Department G2—Aquatic Chemistry, Federal Institute of Hydrology, Am Mainzer Tor 1, 56068 Koblenz, Germany; 2 Thermal and Separation Processes, Hamburg University of Technology, Eißendorfer Straße 38, 21073 Hamburg, Germany; 3 Environmental Chemistry, University of Koblenz-Landau, Fortstraße 7, 76829 Landau, Germany; 4 Department of Chemistry, Technical University of Munich, Lichtenbergstraße 4, 85747 Garching, Germany; 5 Department of Chemistry, University of Gujrat, Gujrat, 50700 Pakistan

**Keywords:** Nanoparticles, Cloud point extraction, Colloids, Coacervate-based techniques, Enrichment, Sample preservation

## Abstract

Coacervate-based techniques are intensively used in environmental analytical chemistry to enrich and extract different kinds of analytes. Most methods focus on the total content or the speciation of inorganic and organic substances. Size fractionation is less commonly addressed. Within coacervate-based techniques, cloud point extraction (CPE) is characterized by a phase separation of non-ionic surfactants dispersed in an aqueous solution when the respective cloud point temperature is exceeded. In this context, the feature article raises the following question: May CPE in future studies serve as a key tool (i) to enrich and extract nanoparticles (NPs) from complex environmental matrices prior to analyses and (ii) to preserve the colloidal status of unstable environmental samples? With respect to engineered NPs, a significant gap between environmental concentrations and size- and element-specific analytical capabilities is still visible. CPE may support efforts to overcome this “concentration gap” via the analyte enrichment. In addition, most environmental colloidal systems are known to be unstable, dynamic, and sensitive to changes of the environmental conditions during sampling and sample preparation. This delivers a so far unsolved “sample preparation dilemma” in the analytical process. The authors are of the opinion that CPE-based methods have the potential to preserve the colloidal status of these instable samples. Focusing on NPs, this feature article aims to support the discussion on the creation of a convention called the “CPE extractable fraction” by connecting current knowledge on CPE mechanisms and on available applications, via the uncertainties visible and modeling approaches available, with potential future benefits from CPE protocols.

## Introduction

Coacervate-based techniques are intensively used in environmental analytical chemistry to enrich and extract different kinds of analytes. Most methods, also presented in several reviews (e.g., [1]), focus on the total content or the speciation of inorganic and organic substances. Size fractionation is less commonly addressed. Within coacervate-based techniques, the cloud point extraction (CPE) is characterized by a phase separation of non-ionic surfactants dispersed in an aqueous solution when the respective cloud point temperature (*T*_c_) is exceeded. In the 1970s, Watanabe and co-workers presented a first series of studies describing methods capable to enrich metals from different matrices (e.g., [2]), and in 2009 Liu and co-workers published the first articles focusing on the extraction and pre-concentration of engineered nanomaterials by CPE [3].

In this context, this feature article raises the question: May CPE in future studies serve as a key tool to (i) enrich and extract nanoparticles (NPs) from complex environmental matrices prior to analyses and to (ii) preserve the colloidal status of unstable environmental samples? With respect to engineered NPs, a significant gap between environmental concentrations (ng/L) and size- and element-specific analytical capabilities is still visible (μg/L or mg/L) [4]. CPE may support efforts to overcome this “concentration gap.” Most colloidal environmental systems known (e.g., waste water, sediment pore water, or surface water) are unstable, dynamic, and prone to changes of the environmental conditions during sampling and sample preparation (e.g., impacted by the oxygen concentration). Even if samples are transported to the laboratory and analyzed as fast as possible, it remains questionable if the state “zero” (representing the in situ) status can be captured. This “sample preparation dilemma” in environmental nanometrology is often, in the absence of an adequate convention, ignored. An example for the impact of such a sample preparation convention is the operationally defined 0.45-μm filtration cut-off for the “dissolved fraction” in combination with an acidification of the samples to preserve the total metal content. When this convention was defined in the 1930s (with a focus on bacteria and the membranes available), it was already known that the term “dissolved” is wrong, since the cut-off allows colloids to pass the membrane. Nevertheless, creating this convention enabled the comparability of data worldwide. The authors are of the opinion that an operationally defined convention for size fractionation is also needed for NPs/colloids in unstable environmental or ecotoxicity test matrices. Such a convention could be supported by CPE-based methods. Focusing on NPs, this feature article sparks the discussion on the creation of a convention called the “CPE extractable fraction” by connecting (i) current knowledge on CPE mechanisms and on available applications, via (ii) the uncertainties visible and modeling approaches, with (iii) potential future benefits from CPE protocols.

## Cloud point extraction

### Phase separation and CPE theory

Referring to Nazar et al. 2011, the aqueous solutions of non-ionic surfactant micelles exhibit thermoreversible phase separation phenomena on heating/cooling through a *T*_c_. As explained by the authors, *T*_c_ can be considered to be the upper stability temperature for the diluted dispersed micellar phase. By approaching *T*_c_ from lower temperatures, intermicellar interactions increase and clouding becomes visible. The previously transparent micellar dispersions scatters now light efficiently. As explained by the authors, above *T*_c_ micelle attractions dominate, and this generates a macroscopic separation in the diluted and the concentrated surfactant phases [5]. Furthermore, the authors describe that the phase behavior of certain non-ionic surfactants in aqueous solutions can readily yield a surfactant-rich phase with a reduced volumes above *T*_c_ and that from an economic and environmental point of view, the process is simple, cheap, highly efficient, and less environmentally hazardous compared to approaches containing organic solvents [5].

**Fig. 1:**
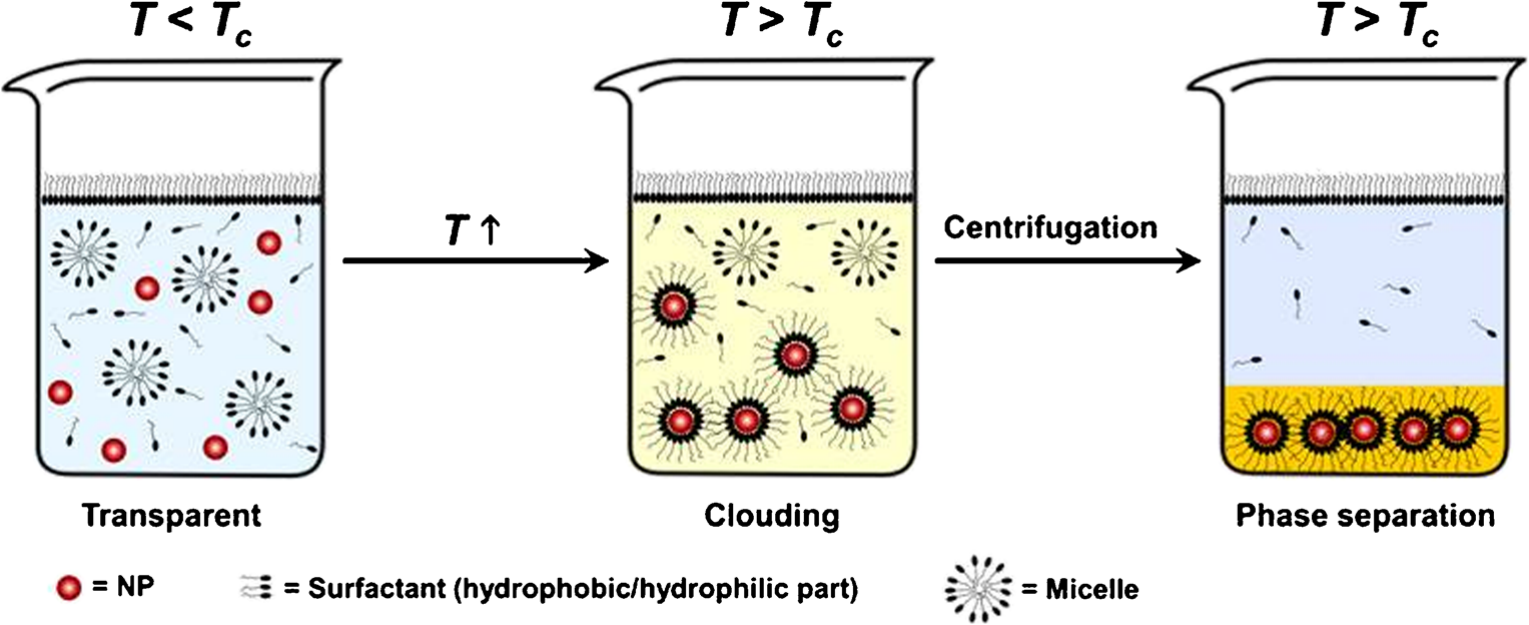
Schematic representation of the surfactant-mediated phase separation process

Several authors proposed mechanisms based on the phase separation phenomenon. It was suggested that above the *T*_c_, a temperature-induced dehydration process occurs in the external layer of the micelles of the non-ionic surfactants. This is due to a decrease in the dielectric constant of water with an increase in temperature that reduces the interaction between the hydrophilic portion of surfactant and water [6]. By breaking hydrogen bonds between water molecules and hydrophilic chains of the surfactant with rising temperature, surfactant micelles become more hydrophobic and phase separation starts (Fig. 1).

### Mechanisms

As previously published in several studies, it is proposed that for non-ionic surfactants the core is surrounded by a mantle of aqueous hydrophilic chains, and solubilization may occur in both (core and mantle), e.g., [7, 8]. As detailed by Purkait et al. 2004: The relative amount of solubilization in these two regions of non-ionic micelles depends on the ionic character of the solubilizate, and the non-ionic surfactants appear relatively to be more hydrophobic at higher temperatures due to an equilibrium shift that favors dehydration of the ether-groups. As *T*_c_ is approached, the solubilization of non-polar solubilizates increases. For polar solubilizates, solubilization decreases caused by dehydration of the nonpolar chains accompanied by increased coiling. In contrast, it is described by the authors that nonpolar compounds are solubilized in the core of micelles, while polar solubilizates are located on the mantle. The authors state that both of the mentioned temperature effects are also consistent with considerations on variations of the space available for the solubilized molecules in the micelles [8].

## CPE and fractionation

### Applications and uncertainties in enrichment and sample preservation

As mentioned before, Liu et al. were the first to apply CPE to concentrate and separate a variety of nanomaterials, like CdSe/ZnS quantum dots, Fe_3_O_4_, TiO_2_, Ag and Au NPs, C_60_ fullerenes, and single-walled carbon nanotubes in aqueous samples [3]. Based on these early findings, CPE methods were worldwide designed to enrich and detect metal-containing NPs in complex matrices. Due to the variety of materials (including also core-shell particles), matrices, and CPE-protocols, a thorough validation for each specific application is inevitable to implement adequate procedures and to identify preparation steps and factors that influence and (potentially) bias the results. To support a better understanding on the current scientific status, in this section, a brief selection of methods, sorted by the target analyte and focusing on metal-containing NPs, is presented.

In different publications, Hartmann et al. optimized the CPE procedure for an enrichment of AuNPs and different AgNPs (including several particle coatings) from aqueous samples in the presence of the respective dissolved fractions [9, 10]. Using Triton X-114 as surfactant, an enrichment factor of 80 from an initial 40-mL sample volume was achieved. Besides different coatings and NP concentrations, the influence of matrix constituents on the extraction was examined. It was shown that the CPE protocols were capable to address different concentrations and a variety of coatings also in the presence of high contents of inorganic salts, colloids, or organic matter. Moreover, the particle size distribution determined using transmission electron microscopy (TEM) was constant for several hours. However, the results also showed that the extraction efficiency can be dependent on the particle size (e.g., 101 % for 2 nm and 52 % for 150 nm AuNPs) as well as that the coating (e.g., bovine serum albumin) possibly lowers the extraction efficiency. To increase traceability, an exemplary protocol to enrich Ag NPs developed by Hartmann et al. [10] is presented: 40 mL of a Ag NPs containing aqueous sample was mixed with 1.0 mL of saturated ethylenediaminetetraacetic acid disodium salt solution, 400 μL of 1 M sodium acetate, 100 μL of 1.25 M acetic acid, and 1 mL of 10 % (*w*/*w*) Triton X-114. The mixture was incubated at 40 °C for 30 min and centrifuged for 12 min at 4427 g to enhance phase separation. Afterwards, the samples were cooled in an ice bath for 5 min to increase phase separation. The aqueous supernatant was removed by decanting. The remaining surfactant-rich phase containing the enriched Ag NPs was dissolved in 100 μL ethanol and subjected to electrothermal atomic absorption spectrometry (ET-AAS) measurement.

Comparably, CPE procedures were established for CuO NPs and ZnO with enrichment factors of 100 and 220 and extraction efficiencies of almost 90 and 64–123 %, respectively [11, 12]. In the case of ZnO NPs, TEM and UV/Vis measurements revealed that particle size and shape were stable over 2 months of storage. By investigating the potential impact of several parameters (e.g., of natural organic matter (NOM)) on the extraction efficiency of CuO NPs, Majedi et al. demonstrated that an interfering effect of adsorbed NOM can be lowered by adding H_2_O_2_ [13]. To simultaneously address Ag, Au, and Fe_3_O_4_ NPs in a single CPE protocol, Tsogas et al. applied a sequential back-extraction and dissolution method of the extracted NPs with recoveries between 74 and 114 % [14]. The method was adapted to complex environmental matrices considering the challenges of co-extracted dissolved species.

Although some of the studies mentioned above addressed the effect of particle size on the extraction efficiency during CPE, it is of utmost importance to increase our understanding on size-resolved extraction efficiencies in order to define precisely the upper and lower particle size cut-off and to properly describe the “CPE extractable size fraction.” So far, the storing capabilities of some CPE protocols are very promising (e.g., [12]). However, to increase the reliability, further investigations concerning size preservation capabilities of CPE for mid- and long-term storing purposes have to be undertaken for other engineered NPs and colloids. To gain a better understanding on the natural occurring processes, the relevant environmental concentration levels for engineered NPs (ng/L range) together with a high colloidal and particle background (mg/L range) should be addressed in the future.

### Detectors and combination with size-specific methods

Together with the colloids and particles, various other substances (e.g., NOM) are known to be present in the extracted phase [15]. Hence, like for almost any other method, the use of selective and sensitive determination techniques is essential for quantitative and qualitative NP analysis due to a limited species selectivity of CPE. ET-AAS is a quantitative, sensitive, and robust element-specific direct detection method for metal-containing NPs. The ET-AAS provides a good matrix tolerance by gradual temperature programs and an effective background correction option. Plasma-based mass spectrometer systems provide a sensitive multi detection opportunity in combination with CPE. However, in contrast to ET-AAS, the samples have to be diluted or digested prior to analyses, unless an ET oven is coupled to the spectrometer [16]. Even though size determination of NPs in environmental relevant concentrations (sub-ng/L up to μg/L) is still challenging, several options are available as a size-specific method after enriching the NPs by CPE: Microscopy-based methods, like TEM, are common tools to determine the size distributions of (nano)particles [9]. Advantages of the methods are the high resolution and a direct data evaluation. Drawbacks are limitations with respect to the particle concentration and the time required for data processing. CPEs enriching capabilities, prior to microscopy-based analyses, have the potential to improve the data reliability and the overall costs.

Size-specific separation techniques in combination with CPE and inductively coupled plasma-mass spectrometry (ICP-MS) are also promising tools. An advantage of the combination with CPE is, to a certain extent, a consistent matrix influence on the analysis. Examples for separation techniques are field-flow fractionation systems (e.g., asymmetric flow field-flow fractionation). However, in this context, the previously mentioned “concentration gap” between environmental concentration and laboratory methods becomes visible, and the CPE extraction and enrichment capabilities display also in this case a potential solution to overcome this gap. The single particle mode of ICP-MS (spICP-MS) appears to be another option to combine a high selectivity and good sensitivities with satisfying analyses times. Via species separation and fractionation, CPE can help to lower or even eliminate the background signal from dissolved species. Multi-element analysis is not sufficiently supported by currently available spICP-MS systems. New developments on time of flight analyzers in combination with spICP-MS allow simultaneous size determination of various elements and isotopes [17], and again future fractionation and NP enrichment multi-element methods may benefit from CPE.

Taken together, CPE can serve as a powerful enrichment and preservation tool for (engineered) NPs in complex environmental matrices in combination with different element- and size-specific detectors.

### Applications and uncertainties in colloidal/dissolved species separation

As mentioned before, usually CPE protocols are designed to extract and pre-concentrate ionic species, organic substances, or NPs in the surfactant-rich phase. The aqueous phase is usually removed and (if at all) only analyzed to validate the CPE procedure. However, to examine the environmental fate and toxicity of engineered NPs, the information on dissolved fractions should also be taken into account. In some cases, the latter is simply calculated as the difference between the particulate and the total concentration. To the best of the authors’ knowledge, only one publication analyzes the aqueous phase to determine the dissolved fraction obtained by application of the CPE [18]. Therefore, additional efforts and method development are needed to further optimize and validate CPE fractionation approaches for determining mass balances.

Regarding natural colloids, no optimized CPE protocol is available, yet. Natural organic colloids (e.g., humic substances) are so far addressed solely with regard to their influence on the extraction efficiency of engineered NPs [13].

## Modeling

Due to their complex phase behavior, colloidal systems are difficult to model. Therefore, most of the models were based on correlations only. In quantitative structure–property relationship (QSPR) models, the target property is correlated with the compounds’ properties and structures, where a training data set is needed to determine the parameters. As a result, the applicability is often limited to molecules similar to those in the training set. Furthermore, no information on the underlying atomistic phenomena is provided. Nevertheless, QSPR models for surfactants have been introduced for example for critical micelle concentrations, cloud point temperatures, surface tensions, biodegradation potentials, etc. [19]. For CPE, the *T*_c_ is a key property that is a priori unpredictable by any thermodynamic model yet. Even the molecular structures at the cloud point and in the micellar phase are not finally resolved. There is some controversy whether the structure is composed of giant micelles or of small aggregated micelles. However, this discrepancy may have been solved, as branched micelles were found near the cloud point [20]. If coacervate systems are used for the enrichment of compounds, the partition behavior of these compounds between the aggregates (e.g., micelles) and the aqueous phase are another key property. Often, octanol/water partition coefficients are used to approximate partition coefficients in micellar systems. To predict partition behavior in such anisotropic systems, an extension of the thermodynamic model COSMO-RS, named COSMOmic, was introduced [21] and successfully applied to predict micelle/water partition coefficients [22]. Unfortunately, this model cannot take into account structural changes of micelles due to the insertion of solutes and, hence, cannot be applied to NPs that alter the microstructures in the micellar phase. Due to the size of NPs, it is important to understand their interactions with surfactants and the microstructures formed with respect to these interactions. To predict suspension microstructures and phase behavior of micelle-NP systems, a statistical mechanical model was introduced that was found to be in agreement with experimental results of dilute suspensions and could be used to analyze colloidal interactions of polymer-like micelles [23]. Another option to investigate molecular structures is the application of molecular dynamics (MD) simulations. For example, with the aid of these simulations, aqueous systems containing cationic surfactants were recently studied. It was shown that the surfactants form a bilayer around NPs with negatively charged surfaces and monolayers around hydrophobic NPs [24].

In the future, it will be particularly important to understand the microstructures formed in coacervate systems. The *T*_c_, as one of the key properties, can probably only be modeled on a physically sound basis, if the structures at the cloud point and in the colloidal phase are known. Furthermore, the microstructures formed between NPs and surfactants are of high interest. As these particles can have different morphologies, sizes, and coatings, a diverse variety of structures, especially after surface transformations in complex (environmental) matrices, are given. In addition, the large varieties of surfactants and surfactant-like natural compounds have different influences. Moreover, complex mixtures will influence the microstructures and therefore *T*_c_ and extraction efficiencies. To obtain a better understanding of these structures, MD simulations and experimental techniques should be used. As in other fields, a combination of both could help to overcome the limitations of a single method. MD simulations can give a detailed atomistic picture at the cost of computationally demanding simulations. Especially, NP systems can be demanding due to the large size (compared to molecules) of the particles. Although atomistic detail is preferable, the simulations could be speed-up by using coarse-grained models at the expense of losing atomistic details. Once the microstructures are understood, general conclusions may be drawn about the formation of these structures and the influencing factors. With this knowledge, it is then probably possible to introduce a thermodynamic model to predict *T*_c_ and partition behavior of NPs.

## Future perspective: by convention—the CPE extractable fraction

Besides its usefulness to recover and re-use high-value engineered NPs or to remove NPs from industrial waste water, a large number of different CPE protocols applied to numerous scientific questions of different working areas (e.g., the extraction and/or pre-concentration of metal ions, NPs, or organic substances or the speciation of metals and metalloids [1]) make CPE today a powerful tool in environmental analytical chemistry. Against this background, it is remarkable that the chance to combine state of the art multi-element analyses by means of ICP-MS with CPE is still fairly unexploited. As detailed in the mechanisms and the modeling sections, a lack of understanding of basic mechanisms of the fractionation process is still apparent. It should be further clarified, which compounds, in dependence of the protocol applied, are exactly included in the two phases. This is a challenge with respect to the experimental as well as the analytical settings, but is urgently needed to better understand and characterize the size fractionation principles. Prospectively, the development of more universal, but still easily implementable, routine-suitable procedures is desirable to increase the efficiency, reproducibility, and comparability of CPE applications. Automatization of the preparative steps and an online coupling to the detectors might become a solution. If future studies can address the mentioned uncertainties, CPE may become a key enrichment and, even more important, preservation method in environmental nanometrology and colloid sciences. To foster the potential of cost-efficient and simple CPE methods, (i) a better understanding on the basic CPE mechanisms, (ii) an open access data base with varying CPE protocols in complex matrices, and, finally, (iii) models on the micelle formation in complex systems are needed to create a convention on the “CPE extractable fraction.” Even if the previous points appear challenging, the earning would be not less than an international common basis for coordinated environmental monitoring efforts on NPs and colloids. As a surplus, via a common convention to fix the status of dynamic colloidal systems, a higher reliability in environmental nanometrology can be achieved for spectroscopy, mass spectrometry, or microscopy-based analyses.
